# Attentional bias for alcohol cues in visual search—Increased engagement, difficulty to disengage or both?

**DOI:** 10.1371/journal.pone.0228272

**Published:** 2020-01-27

**Authors:** Janika Heitmann, Nienke C. Jonker, Brian D. Ostafin, Peter J. de Jong

**Affiliations:** 1 Verslavingszorg Noord Nederland, Groningen, The Netherlands; 2 Department of Clinical Psychology and Experimental Psychopathology, University of Groningen, Groningen, The Netherlands; Radboud University, NETHERLANDS

## Abstract

Cognitive models emphasise the importance of attentional bias in addiction. However, many attentional bias tasks have been criticised for questionable psychometric properties and inability to differentiate between engagement and disengagement processes. This study therefore examined the suitability of two alternative tasks for assessing attentional bias within the context of alcohol use. Participants were undergraduate students (*N* = 169) who completed the Visual Search Task and Odd-One-Out Task, the latter of which is designed to differentiate between engagement and disengagement processes of attention, at baseline and one week later. Participants also completed baseline measures of alcohol consumption, craving, and alcohol use problems. Internal consistency was adequate for the Visual Search Task index, and weak for the Odd-One-Out Task indices. Test-retest reliability was weak for both tasks. The Visual Search Task index and the disengagement (but not the engagement) index of the Odd-One-Out Task showed a positive association with alcohol consumption. This study was restricted to a non-clinical student sample. The relatively high error rate of the Odd-One-Out Task might have reduced its sensitivity as an index of attentional bias. Both tasks showed some merit as attentional bias measures, and results suggested that attentional disengagement might be particularly related to alcohol use. However, the reliability of the current measures was inadequate. One potential explanation for the low reliability is that non-clinical samples may have weak and unstable attentional biases to alcohol. Future efforts should be made to improve the psychometric qualities of both tasks and to administer them in a clinical sample.

## Introduction

Current cognitive models of addiction emphasise the importance of selective visual attention in the persistence of addiction [1,2]. More specifically, increased attention for substance cues has been associated with the intensity of craving [3,4]. In turn, craving may increase attentional capture of substances, and/or the difficulty to disengage attention from these cues. As a result, people may enter a self-reinforcing bias-craving-bias cycle that may play an important role in the persistence of addiction. These attentional tendencies, known as Attentional Bias (AB), have therefore received a lot of interest in the field of substance use disorders [5]. Related research has resulted in an improved understanding of the disorders and also in the development of new treatment tools (e.g., attentional bias modification trainings). However, these developments also draw attention to the limitations of existing measures as indices of individual differences in AB.

One of the most frequently used paradigms to measure substance-related AB is the visual probe task [6]. Although the visual probe task is widely used, it is not without its critics. First, the reliability of the task in terms of internal consistency and test-retest reliability has been found to be poor [7–9]. A low test-retest reliability is especially problematic when the task is used to measure changes in AB. To account for the low reliability of the visual probe task and other indirect measures of AB, several studies have tested whether more direct measures of attention by the use of eye-tracking can serve as an alternative [10,11]. However, although eye-tracking can measure overt attention shifts (responding by directly moving and focussing the gaze on a target), it does not measure covert attention shifts (responding by seeing something peripherally without directly focussing the gaze on a target). Eye-tracking might be a valuable addition to this field of research, but as also encouraged by other researchers [12] a task that can reliably measure the influence of covert attention shifts remains also desirable (see for example attempts to improve the reliability of the visual probe task, [13]).

Second, it has been questioned whether the presentation of just one pair of stimuli, such as in the visual probe task, can adequately reflect the complexity of real-life substance use relevant situations in which a person is constantly surrounded by multiple stimuli [14]. To make attentional bias tasks more compatible with the complexity of real-life substance use situations, it seems essential to more closely mimic this complexity by the use of a more complex stimulus configuration. One paradigm with a more complex stimulus configuration is the flicker-induced change blindness paradigm [15], which has been adapted to measure AB towards alcohol cues. Using this paradigm, several studies have found differences in AB between heavy and social/light drinkers [16,17]. However, the reliability of this task remained unexplored.

Another promising example of a paradigm with a more complex stimulus configuration is the visual search paradigm. In this paradigm participants identify a target stimulus among a series of distractors [18]. The visual search paradigm has been successfully applied in the context of anxiety disorders [19], eating disorders [20], and pain disorders [21]. Yet, this paradigm has been largely ignored in the field of addiction, with the exception of one promising study assessing AB in smokers [22]. The aim of the current study is therefore to examine the potential of the visual search paradigm as an index of AB in the context of alcohol use.

There are two sub-types of the visual search paradigm, which differ with respect to the indices of AB they assess. In the first type of task, often referred to as the Visual Search Task (VST), individuals actively search for a target stimulus in a matrix of distractors [23]. In the case of an AB for alcohol, participants are expected to be faster in detecting an alcohol cue in an array of non-alcohol distractors than detecting a non-alcohol cue in an array of alcohol distractors. Due to the typical methods of the task and the scoring procedure, interference effects might be due to either automatic orientation towards an alcohol cue (i.e., attentional engagement), maintaining attention on alcohol cues due to a difficulty to disengage (i.e., attentional disengagement), or both. However, similar to the visual probe task and the flicker-induced change blindness task, the VST is not able to differentiate between these different processes of attention.

The second sub-type of the visual search paradigm, called the Odd-One-Out Task [18,24], is able to deliver two indices that have been supposed to reflect attentional engagement and disengagement, respectively. In the OOOT, individuals indicate whether images in a matrix are from the same category of images or whether there is an odd-one-out (i.e., target image). The OOOT includes trials in which a neutral target is presented among neutral distractors drawn from a different category. This trial type can serve as a personal baseline of how long it generally takes to find a target between distractors, making it possible to calculate separate indices for increased engagement and difficulty to disengage. That is, attentional engagement is inferred from the relative speeding to correctly respond that an odd-one-out is present when an alcohol stimulus is presented among neutral distractors, compared the time taken to a correct response when a neutral stimulus is presented among neutral distractors (from a different neutral category; cf. [20]). Difficulty to disengage from alcohol cues is inferred from the relative slowing to correctly respond that an odd-one-out is present when a neutral stimulus is presented among alcohol distractors, compared the time taken to a correct response when a neutral stimulus is presented among neutral distractors (from a different neutral category; cf. [20]).

The differentiation between attentional engagement and disengagement might be relevant, as earlier research in different psychopathologies indicated that different disorders might be characterised by one of these attentional processes or both [20,24]. More detailed knowledge about these attentional processes might not only be essential for the general understanding of the disorder, but particularly important when it comes to treatment indications, for example in terms of testing the efficacy of attentional bias modification interventions that can be tailored to directly target the relevant process to improve treatment outcome. Given that earlier studies in substance use have mainly used tasks that were not able to differentiate between increased engagement and difficulty to disengage, such as the visual probe task, the interpretation of attentional indices may have contributed to the inconsistency of findings across studies [25]. A task, such as the OOOT, that is able to make this differentiation seems therefore relevant to further disentangle the AB processes that contribute to substance use.

The aim of the current study was to investigate the potential of both the VST and the OOOT as an index of AB for alcohol cues. As a first step, we examined the tasks’ reliability. Having strong internal consistency and test-retest reliability would provide confidence in the tasks’ ability to capture individual differences in AB and its changes. As a next step, we tested the (predictive) validity of the tasks by examining the association between their indices of AB with self-reported alcohol consumption (i.e., quantity and frequency), craving, and alcohol use problems. In particular, we were interested in whether the two underlying processes of AB assessed by the OOOT–attentional engagement and disengagement–were differentially related to alcohol use. We expected that if the VST and the OOOT are adequate methods for assessing individual differences in AB for alcohol cues, the AB measures will be positively associated with the drinking outcome measures.

## Method

### Participants

Participants were 169 undergraduate Psychology students (18.3% male) with a mean age of 20.55 years (*SD* = 2.80; Age and gender were unknown for one participant) from the University of Groningen. Initially, 182 participants participated in the study, but 13 participants dropped out after the first assessment. Therefore, their data were not included in the current analyses.

### Material

#### Alcohol use and craving

The quantity of used alcohol, the frequency of use, and craving for alcohol were assessed with the Measurements in Addiction for Triage and Evaluation Questionnaire (MATE-Q; [26]). The quantity of the past 30 days was indexed by the amount of standard glasses of alcohol consumed on a typical Monday, Tuesday, Wednesday, etc. The answers per week were multiplied by four to estimate the consumed standard units of alcohol over the past month. To index the frequency of use, participants were asked to indicate on how many of the last 30 days they consumed alcohol. The Obsessive-Compulsive Drinking Scale (OCDS5) of the MATE-Q was used to assess general craving. It consists of five items measuring the desire for alcohol in the past seven days. Participants indicated their answers on a 5-point Likert scale. Reliability of the OCDS5 as estimated with Cronbach’s alpha was poor (α = .49). This seemed to be related to item four of the questionnaire (i.e., How much of an effort do you make to resist these thoughts or try to disregard or turn your attention away from these thoughts as they enter your mind when you’re not using?), which among clinicians is known to be difficult to understand. When excluding this item, Cronbach’s alpha was acceptable (α = .75). In addition to the OCDS5, state craving was assessed by asking participants to indicate their amount of craving for alcohol directly before performing the VST and the OOOT on a Likert scale from 1 (no craving) to 7 (extreme craving).

#### Alcohol use problems

Problems with alcohol use were assessed with the Rutgers Alcohol Problem Index [27] consisting of 18 questions. Participants indicated on a 5-points Likert scale, ranging from ‘never’ to ‘very often’, how often they had experienced the described situation in the past. Cronbach’s alpha of this questionnaire was good (α = .85).

#### Visual Search Task (VST)

**Fig 1:**
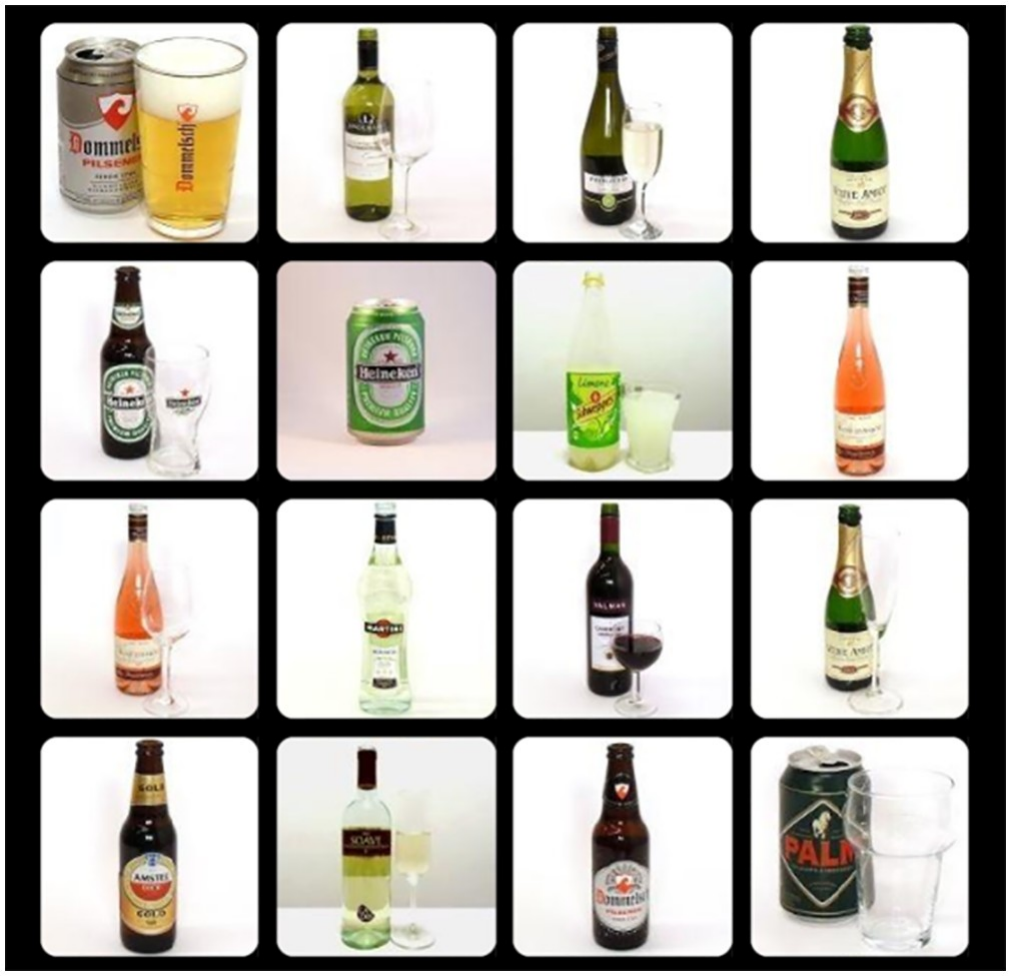
Example of an alcohol distractors trial of the VST.

Each trial of the VST started with a red fixation cross appearing in the centre of the screen [23]. Participants located the mouse cursor on the cross to start the trial, after which a 4 x 4 matrix of 16 images (500 x 500 pixels) appeared. Fifteen of the 16 images belonged to the same category of images, whereas one image was different (i.e., target stimulus). Participants clicked as quickly and as accurately as possible on the target image of the search category that was named before the task started. The images belonged to one of the following two categories: alcoholic drinks or non-alcoholic drinks. The next trial appeared after a 1000 ms inter-trial interval. The task was divided into four blocks, two blocks of which the target was an alcohol image in an array of non-alcoholic drinks distractors (i.e., *alcohol target trials*), and two blocks in which the target was a non-alcoholic drink image that had to be found among alcohol distractors (i.e., *alcohol distractors trials*). Each block consisted of 18 trials, and per block the target was presented in all possible positions in a random order. The blocks were presented alternately, and the first block was chosen randomly for each participant (Fig 1). AB index was calculated by subtracting the mean reaction time of *alcohol target trials* from the mean reaction time of *alcohol distractors trials*. Higher positive scores reflected stronger AB for alcohol.

#### Odd-One-Out Task (OOOT)

**Fig 2:**
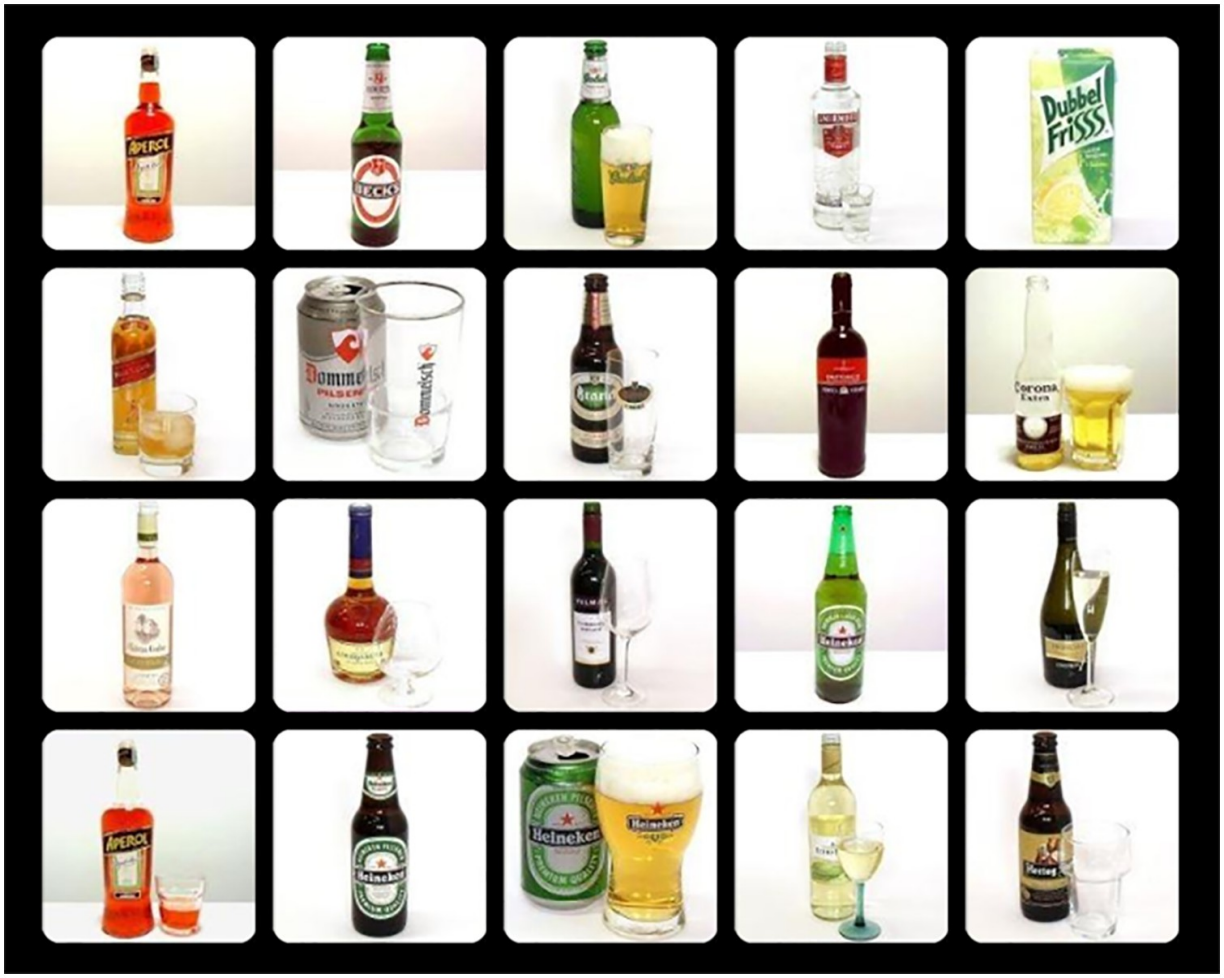
Example of an alcohol distractors trial of the OOOT.

In the OOOT, participants focused their attention on a red fixation cross in the centre of the screen for 500 ms [18]. Afterwards a 4 x 5 matrix of 20 images (500 x 500 pixels) appeared, and participants indicated whether an odd-one-out image (i.e., target stimulus) was present. Responses were given by pressing the ‘0’ (no odd-one-out) or ‘1’ (yes, odd-one-out present) button of the keyboard. Participants had a maximum of 10 seconds to respond, and the task was divided into three blocks of 24 trials each. The order of trials was random, and for each block the target randomly appeared over the possible positions, but never directly above or below the fixation cross. Each image belonged to one of the following three categories: alcoholic drinks, non-alcoholic drinks, or flowerpots. Given these three different categories of images, nine different combinations of trials were possible. For each block, an equal number of trials was included for all trial types without an odd-one-out (6) and all trial types including an odd-one-out (18; Table 1; Fig 2). Attentional engagement (i.e., engagement index) was calculated by subtracting the mean reaction time of the *alcohol target trials* (i.e., alcohol target in either non-alcoholic drinks distractors or flowerpot distractors) from the mean reaction time of the *neutral target in neutral distractors trials* (i.e., non-alcoholic drinks target in flowerpot distractors or flowerpot target in non-alcoholic drinks distractors). Higher positive scores were expected to reflect more attentional engagement with alcohol cues. Difficulty to disengage (i.e., disengagement index) was calculated by subtracting the mean reaction time of the *neutral target in neutral distractors trials* from the mean reaction time of the *alcohol distractors trials* (i.e., non-alcoholic drinks target or flowerpot target in alcohol distractors). Higher positive scores reflected more difficulty to disengage attention from alcohol cues.

**Table 1:** Type and amount of trials in the Odd-One-Out Task (OOOT).

Trial type			Trials per block
1.	Alcohol (20)		2
2.	Non-alcoholic drinks (20)		2
3.	Flowerpot (20)		2
	**Target**	**Distractors**	
4.	Alcohol (1)	Non-alcoholic drinks (19)	3
5.	Alcohol (1)	Flowerpot (19)	3
6.	Non-alcoholic drink (1)	Alcohol (19)	3
7.	Flowerpot (1)	Alcohol (19)	3
8.	Non-alcoholic drink (1)	Flowerpot (19)	3
9.	Flowerpot (1)	Non-alcoholic drinks (19)	3

Number of presented images per trial is given in parentheses. Trial numbers 4 and 5 (i.e., alcohol target trials), trial numbers 6 and 7 (i.e., alcohol distractors trials) and trial numbers 8 and 9 (i.e., neutral target in neutral distractors trials) were included in the current analyses.

#### Stimuli

Both tasks used the same images of alcoholic drinks and non-alcoholic drinks [28,29]. The OOOT additionally contained images of flowerpots [30]. In total, 90 images were used—30 different images per category (alcoholic drinks, non-alcoholic drinks, and flowerpots). The images were randomly drawn in each trial.

### Procedure

The study was approved by the ethical committee of the psychology faculty of the University of Groningen, and data was collected from October to November 2016 in a quiet laboratory. On arrival, participants provided informed consent. They started with indicating their state craving, and thereafter continued with either the OOOT or the VST. The order of the two tasks was counterbalanced. After finishing both tasks, participants filled in the questionnaires. In order to assess test-retest reliability participants returned to the laboratory after exactly one week. At the second visit, participants again completed both tasks in the same order as the first time. After the completion of the study, all participants were debriefed. Participants received course credits or financial compensation in return for their participation.

### Analyses

To investigate the internal consistency of both tasks, the split-half method was used to calculate Spearman-Brown coefficient between the first half and the second half of the baseline task. To account for a possible learning effect throughout the task a second method to calculate Spearman-Brown coefficient was used by distributing the trials alternately to one of two subsets. The first trial of one particular trial type was randomly allocated to either of the subsets. Split-half reliability was tested for the indices as well as for the trial types of both tasks. The test-retest reliability was also calculated for the trial types and for the indices using Pearson correlations. The estimates for the internal consistency and test-retest reliability were characterised as weak (*r*<.5), adequate (.5 ≤ *r* < .8), or good (*r* ≥ .8) based on commonly reported thresholds [31]. As a second step, repeated measures analyses of variance (RM-ANOVA) were conducted to test whether the performance was stable over time, with trial type (i.e., *alcohol target trials* and *alcohol distractors trials*, and *neutral target in neutral distractors trials* for the OOOT) as independent variable and time (baseline and post-test) as dependent variable. To test to the extent to which alcohol consumption (i.e., quantity and frequency) as assessed with the MATE-Q, general craving as indexed with the OCDS5, and alcohol use problems as assessed with the RAPI-18 were associated with AB as measured with both tasks, bivariate correlational analyses were computed. Consistent with previous research testing the relationship between cognitive performance measures and alcohol use/problems [32], this part of the analyses excluded the data of participants (*n* = 20) who reported that they did not drink alcohol during the last 30 days.

Based on power analyses prior to the study, we aimed for a sample size of at least 150 participants to be able to detect a small to moderate correlation (*r* = .20) with a power of 80% at an *alpha* level of 0.05. The current sample (*n* = 169) provided 84% power to detect a small-moderate correlation at an *alpha* of 0.05.

## Results

### Data preparation

#### Visual Search Task (VST)

Participants scoring 3*SD*’s below the mean percentage correct answers (baseline < 97.53%; post-test < 98.09%) were removed (baseline *n* = 5; post-test *n* = 2), because high numbers of incorrect responses might indicate non-serious participation. In line with Hollitt and colleagues [20], as a next step, incorrect responses were excluded from the analyses (baseline 1.8%; post-test 1.8%). No reaction times were below 200 ms, which were considered anticipation errors. Finally, outliers were calculated based on participants’ average response time per type of trial. Trials scoring 3 *SD*’s below or above participants’ average response time were removed. This resulted in deleting another 1.83% of trials from the baseline and 1.79% of trials from the post-test.

#### Odd-One-Out Task (OOOT)

Identical with the procedure for the VST, participants scoring 3*SD*’s below the mean percentage correct answers (baseline < 82.40%; post-test < 88.00%) were removed (baseline *n* = 2; post-test *n* = 2). As a next step, incorrect responses (i.e., participants indicated that there was no odd-one-out although an odd-one-out was present) were excluded from the analyses (baseline 20.00%; post-test 13.8%). Further examination indicated that the highest number of errors were made in *alcohol distractors trials* when a non-alcoholic drink image was the target (baseline 42.60%; post-test 31.20%), and in *alcohol target trials* in which non-alcoholic drinks images were the distractors (baseline 32.00%; post-test 25.00%). At baseline, participants made significantly more errors when non-alcoholic drinks were used as contrast category compared with the number of errors that were made when flowerpots were used as contrast category (*t*(332) = 13.28, *p* <.001; *t*(332) = 8.71, *p* <.001, respectively for alcohol distractors trials and alcohol target trials). There were no significant differences concerning the number of errors for neutral target in neutral distractors trials (*t*(332) = 1.36, *p* = .174). Anticipation errors (reaction times < 200 ms) were removed from the analyses (baseline 1 trial; post-test 8 trials). No further outliers based on participants’ average response times per type of trial, following the 3*SD*’s rule, were detected.

### Descriptive statistics

The average quantity of drinks consumed during the last 30 days was 47.03 (*SD* = 59.73), and the mean number of days in which alcohol was consumed during the last 30 days was 4.64 (*SD* = 4.58). General craving for alcohol, as measured with the MATE-Q, was 7.05 (*SD* = 2.23)–a value under the critical cut-off of 12 for pathological craving [26], indicating that the current sample had on average a non-pathological level of craving. On an individual level, five participants had a score larger than or equal to the cut-off for pathological craving. The ratings on state craving indicated a generally low level of craving for alcohol directly before the task (*M* = 1.44, *SD* = 0.95). General craving of the past seven days was significantly related with state craving before the tasks (*r* = .37, *p* < .001). Alcohol use problems were in line with normal values of a nonclinical sample (*M* = 8.80, *SD* = 7.54; [27]).

For the VST, the mean index of AB was 375.09 ms (*SD* = 580.56) at baseline, and 286.73 ms (*SD* = 478.35) at post-test. At baseline, the mean of the engagement index of the OOOT was -516.34 ms (*SD* = 420.92), and the mean for the disengagement index was 823.19 ms (*SD* = 537.84). At post-test, the mean of the engagement index was -571.49 ms (*SD* = 444.06), and of the disengagement index 838.84 ms (*SD* = 518.86). Table 2 shows the mean reaction times of all trial types. See the supporting information for the correlations between the VST index and both OOOT indices (S1 Table).

**Table 2:** Alcohol target trials, alcohol distractors trials and neutral target in neutral distractors trials as measured with the Odd-One-Out Task and the Visual Search Task at baseline and post-test.

Baseline				Post-test		
	Alcohol target trials	Alcohol distractors trials	Neutral target in neutral distractors trials	Alcohol target trials	Alcohol distractors trials	Neutral target in neutral distractors trials
OOOT	2524 (590)	2831 (760)	2008 (486)	2178 (518)	2446 (620)	1607 (335)
VST	2997 (642)	3372 (794)	-	2748 (543)	3035 (641)	-

Means and standard deviations are given in ms.

### Internal consistency of attentional bias measures

#### Visual Search Task (VST)

Internal consistency of each of the trial types was good (Table 3). Internal consistency between the first half and the second half of trials for the AB index at baseline was .57. When trials were alternately distributed to either of the two subsets, Spearman-Brown coefficient was .59.

**Table 3:** Internal consistency reported for the split-half and random distribution method per trial type for the Visual Search Task.

Method	Trial type	
	Alcohol target trials	Alcohol distractors trials
Split-half	.83	.84
Random distribution	.90	.83

#### Odd-One-Out Task (OOOT)

Internal consistency of each of the trial types was adequate (Table 4). Spearman-Brown coefficient for the attentional indices when comparing the first half with the second half was -.10 for the engagement index, and .22 for the disengagement index. When trials were alternately distributed to either of the two subsets, internal consistency of the engagement index was .34, and .33 for the disengagement index.

**Table 4:** Internal consistency reported for the split-half and random distribution method per trial type for the Odd-One-Out Task.

Method	Trial type		
	Alcohol target trials	Alcohol distractors trials	Neutral target in neutral distractors trials
Split-half	.65	.66	.60
Random distribution	.77	.75	.81

### Test-retest reliability of attentional bias measures

#### Visual search task (VST)

There was a weak positive correlation between AB at baseline and at post-test that was statistically significant (*r* = .16, *p* = .040). Higher scores of AB at baseline were related to higher scores at post-test. *Alcohol target trials* showed adequate test-retest reliability (*r* = .58, *p* < .01); also *alcohol distractors trials* showed adequate test-retest reliability (*r* = .68, *p* < .01). The RM-ANOVA showed that there was a significant effect of time (*F*(1, 161) = 114.22, *p* < .01, η^2^ = .42), and trial type (*F*(1, 161) = 67.34, *p* < .01, η^2^ = .30). No significant interaction was found (*F*(1,161) = 2.57, *p* = .111, η^2^ = .01), indicating similar temporal changes for both trial types. Paired sample *t*-tests were conducted to indicate whether both trial types differed significantly when comparing baseline with post-test outcomes. There was a significant difference from baseline to post-test for *alcohol target trials* (*t* (161) = 5.81, *p* = <.001), and *alcohol distractors trials* (*t* (161) = 7.32, *p* <.001). Participants became faster from baseline to post-test for both trial types (see Table 2 for the related means). The index of AB did not differ between baseline and post-test (*t* (161) = 1.60, *p* = .111).

#### Odd-One-Out task (OOOT)

There was no statistically significant relation between the baseline and post-test engagement indices (*r* = .09, *p* = .257). The baseline and post-test disengagement indices showed a weak positive correlation (*r* = .23, *p* = .003). Participants showing more difficulty to disengage from alcohol cues at baseline, also showed more difficulty to disengage at post-test. A*lcohol target trials* showed adequate test-retest reliability (*r* = .45, *p* < .001); *alcohol distractors trials* showed adequate test-retest reliability (*r* = .52, *p* < .001). Also *neutral target in neutral distractors trials* showed adequate test-retest reliability (*r* = .54, *p* < .001). RM-ANOVA showed that there was a significant main effect of time (*F*(1, 165) = 125.71, *p* < .001, η^2^ = .43), and a significant main effect of trial type (*F*(2, 330) = 429.90, *p* < .001, η^2^ = .72). The non-significant interaction between time and trial type (*F*(2, 330) = 0.58, *p* = .563, η^2^ < .01), indicated that the temporal changes were similar for all trial types. T-tests were conducted to indicate whether all trial types differed significantly when comparing baseline with post-test outcomes. There was a significant difference from baseline to post-test for *alcohol target trials* (*t* (165) = 7.86, *p* = < .001), *alcohol distractors trials* (*t* (165) = 7.39, *p* < .001), and *neutral target in neutral distractors trials* (*t* (165) = 12.56, *p* < .001). Participants became faster from baseline to post-test for all three trial types (see Table 2 for the related means). There was no difference between baseline and post-test for the indices of AB (engagement index: *t* (165) = 1.11, *p* = .257; disengagement index: *t* (165) = -0.26, *p* = .795).

### Relation between attentional bias measures and outcome measures

#### Visual Search Task (VST)

To examine whether the AB index of the VST was related with alcohol consumption, craving and/or alcohol use problems, correlational analyses were conducted. At baseline, AB showed a weak but significant relationship with the frequency of alcohol use (*r* = .20, *p* = .018), indicating that drinking more regularly was associated with higher scores of AB. All other correlations with the VST were small and non-significant, see Table 5. Also when the outcome variable craving was constructed without the item leading to poor internal consistency, the correlations with AB remained small and non-significant.

**Table 5:** Correlations between attentional bias indices as measured with the VST and the OOOT and the outcome measures.

	Quantity	Frequency	Craving	Alcohol use problems
VST AB	-.04	.20*	.01	-.02
OOOT E	-.02	-.01	-.02	-.03
OOOT D	.18*	.18*	.02	.09

VST AB = index of attentional bias measured with the Visual Search Task; OOOT E = index of engagement as measured with the Odd-One-Out Task; OOOT D = index of disengagement as measured with the Odd-One-Out Task;

*p< .05.

#### Odd-One-Out task (OOOT)

To investigate whether the indices of the OOOT were related with alcohol consumption, craving, and/or alcohol use problems, correlational analyses were conducted (Table 5). We found that specifically the disengagement index showed an association with the quantity of used alcohol (*r* = .18, *p* = .028) and with the frequency of consumption (*r* = .18, *p* = .026), although both associations were weak and only evident for alcohol consumption but not for craving or alcohol use problems (see supporting information for the results when including the non-drinkers to this analysis, S1 File). As for the VST, when the outcome variable craving was constructed with the item leading to poor internal consistency, the correlations with the engagement and disengagement index of the OOOT remained small and non-significant.

### Post-hoc analyses

Post-hoc, two multiple linear regression analyses were performed to examine whether the AB measures derived from the VST and the OOOT would have independent associations with alcohol consumption (i.e., frequency, quantity). The three predictor variables (i.e., VST index, OOOT engagement index, OOOT disengagement index) were centred before they were entered to the model. The assumptions for the linear regression of linearity of residuals, independence of residuals, normal distribution of residuals, and equal variance of residuals were not violated. We found that AB as measured with the VST was independently related to the frequency of alcohol consumption (*β* = .19, *t* = 2.25, *p* = .026), but the OOOT indices were not (engagement: *β* = .01, *t* = 0.11, *p* = .914; disengagement: *β* = .09, *t* = 0.97, *p* = .333). Quantity of alcohol consumption was independently related to the disengagement index as measured with the OOOT (*β* = .21, *t* = 2.12, *p* = .036). There was no independent association between the quantity of alcohol consumption and the engagement index of the OOOT (*β* = .10, *t* = 1.00, *p* = .322), nor with the AB index derived from the VST (*β* = -.07, *t* = -0.80, *p* = .427).

**Fig 3:**
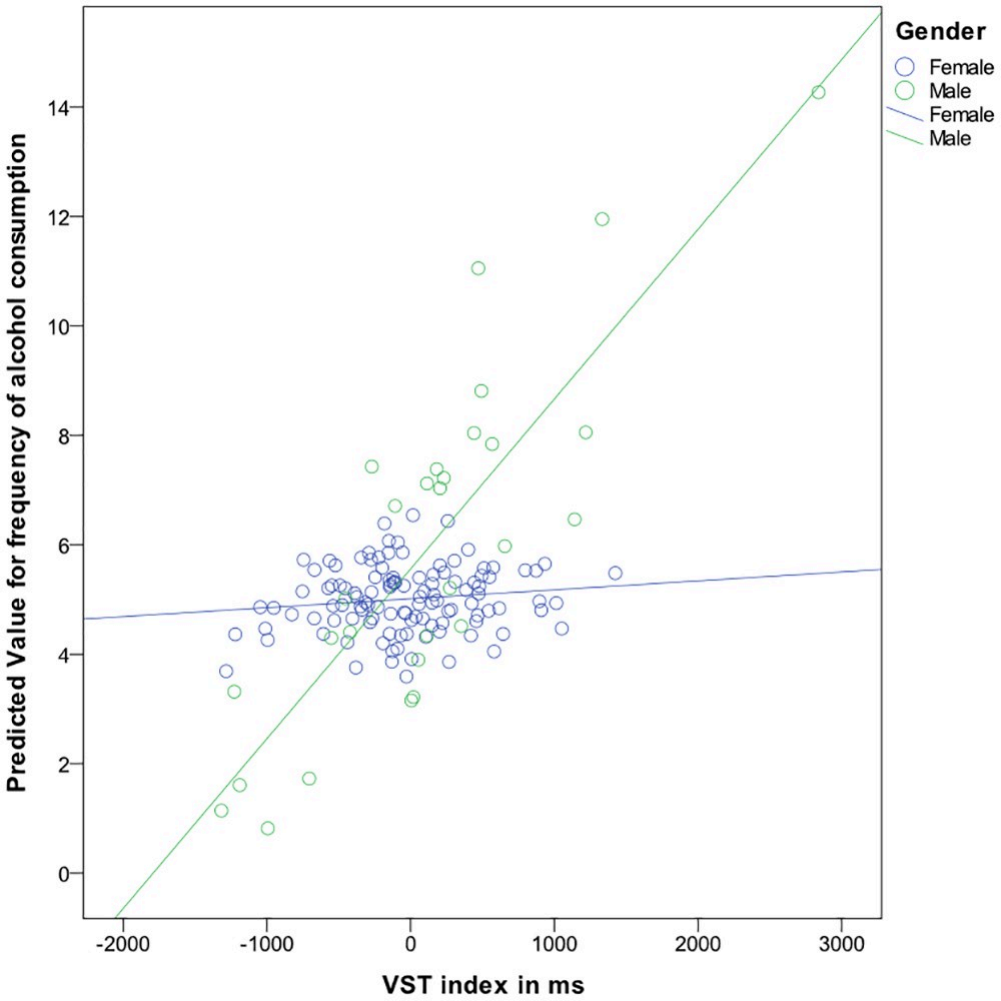
Frequency of alcohol consumption for males and females by AB as measured with the VST.

**Fig 4:**
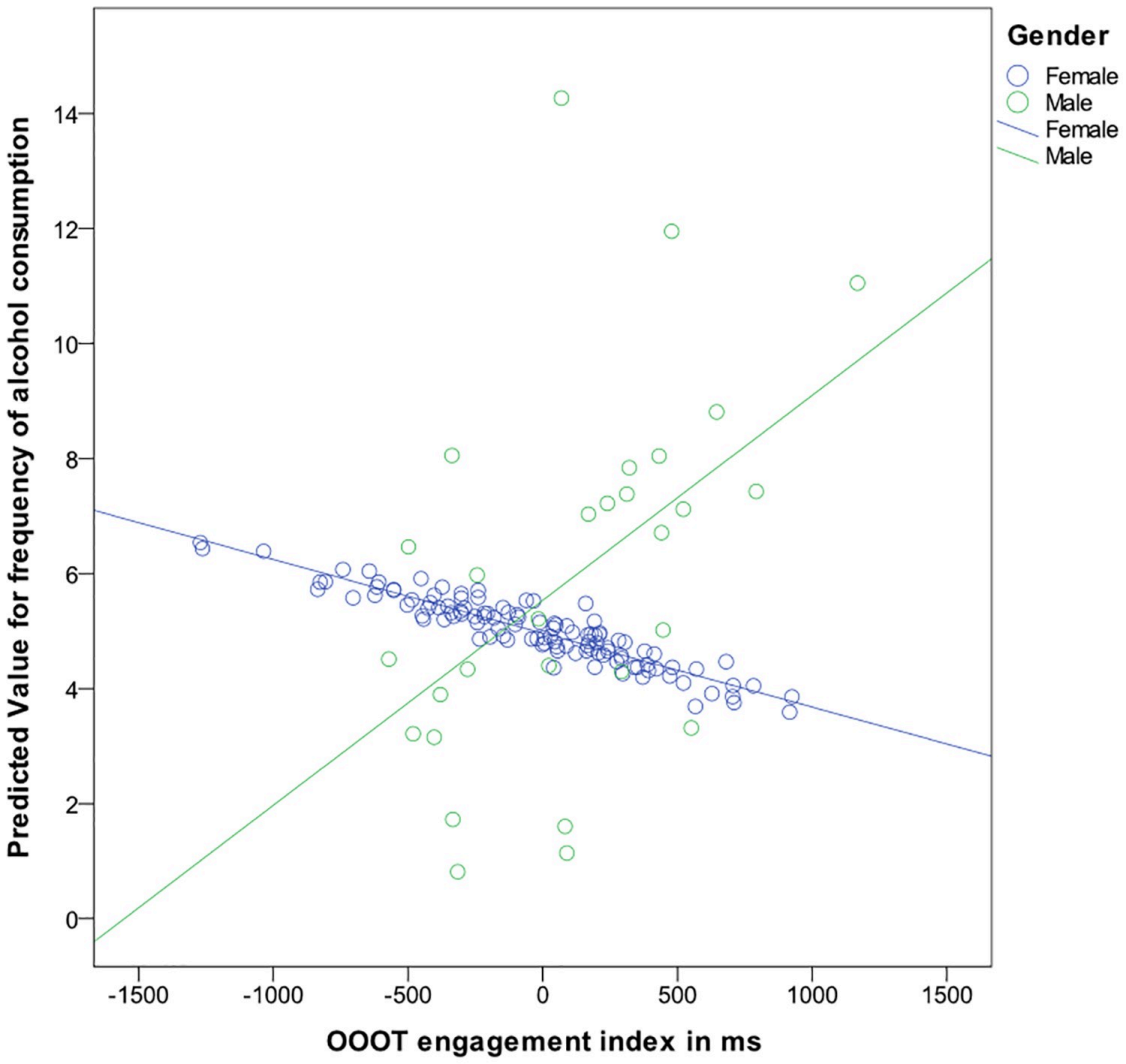
Frequency of alcohol consumption for males and females by attentional engagement as measured with the OOOT.

Given that some previous studies have reported a difference between the association of AB and alcohol consumption with regard to gender we added moderator predictors of gender and the three AB indices to the regression models described above [33,34]. There was no significant independent association with gender in the model when quantity of alcohol consumptions was the dependent variable. However, as can be seen in Table 6, in the model of the predicted frequency of alcohol consumption, the interaction predictor of the VST index and gender, and the OOOT engagement index and gender showed an independent association with the frequency of alcohol consumption (*β* = .24, *p* = .027; *β* = .24, *p* = .021, respectively). For male participants, stronger AB as measured with the VST was associated with more frequent alcohol consumption. For female participants there was no clear pattern (Fig 3). When measured with the OOOT, for male participants more attentional engagement with alcohol cues was associated with more frequent alcohol consumption, whereas for female participants attentional engagement with alcohol cues was not related with more frequent alcohol consumption (Fig 4).

**Table 6:** Linear model of predictors of frequency of consumed alcohol during the past 30 days.

	Beta	t	P-value
Constant		12.54	>.001
VST AB	.04	0.39	.697
OOOT E	-.11	-0.96	.340
OOOT D	.04	0.38	.707
Gender	.01	0.13	.895
VST AB x Gender	.24	2.23	.027
OOOT E x Gender	.24	2.34	.021
OOOT D x Gender	.02	0.18	.861

VST AB = index of attentional bias measured with the Visual Search Task; OOOT E = index of engagement as measured with the Odd-One-Out Task; OOOT D = index of disengagement as measured with the Odd-One-Out Task; R^2^ = .12; adjusted R^2^ = .08.

## Discussion

The current study investigated the psychometric and predictive properties of AB measures derived from two sub-types of the visual search paradigm. In particular, we assessed the internal consistency and the test-retest reliability of the AB measures derived from the VST and the OOOT. Further, the validity of the measures was tested by examining the association between the indices of AB with alcohol consumption (i.e., quantity and frequency), craving, and alcohol use problems. We were especially interested whether it is useful to use a task that is able to differentiate between two underlying attentional processes of AB—engagement and disengagement. The major findings can be summarised as follows: (1) the internal consistency of the AB index as measured with the VST was found to be adequate, and both indices of the OOOT were found to be weak; (2) the test-retest reliability of the AB indices was found to be weak for both tasks; (3) AB for alcohol as measured with both the VST and the disengagement index of the OOOT was found to be (weakly) related with the frequency of alcohol consumption, with the disengagement index of the OOOT also correlating with the number of consumed standard units of alcohol; (4) AB as measured with the VST and the OOOT was non-significantly related with craving and alcohol use problems.

The internal consistency of participants’ responses to particular trial types ranged from adequate to good. Yet, for both the VST and the OOOT the internal consistency of the AB indices was found to be insufficient and smaller than .80, which would be considered good internal consistency. These findings are in line with the internal consistency of AB measures derived from other reaction time tasks [7]. Further, the internal consistency of the VST appeared better than the internal consistency of the OOOT. This difference might be related to the number of errors made in the OOOT, the difference in task complexity, and the unblocked task design (i.e., random order of trial types) of the OOOT [7]. An overall explanation for the low internal consistency of both tasks might be that the current sample consisted of non-clinical participants. Internal consistency is dependent on the population that is examined and there are indications that measures of AB are more reliable in problematic substance users than in ‘healthy’ individuals [35]. That is, in case of a small or absent AB, tasks may be less consistent as they mainly measure noise rather than individual differences in AB. It seems therefore relevant to test the internal consistency of the VST as well as of the OOOT in a clinical sample, as AB is expected to be stronger and more stable in the clinical population. Furthermore, especially the OOOT might be improved by including more trials, using a blocked task design, and by providing participants with feedback to reduce the amount of errors [7]. Another factor that might contribute to the low internal consistency of the AB measures is the use of diverse alcohol stimuli. That is, participants might show a bias toward some alcoholic drinks (e.g., wine) but not to others (e.g., beer; [35]). Therefore, current AB measures might also be improved by using personalised stimuli.

The test-retest reliability of the AB indices of both tasks was found to be weak, which is in line with the low test-retest reliability that has been reported for other AB tasks such as the Stroop task [36]. Although the stability of the AB indices was weak, for both tasks, an adequate test-retest reliability was found for the separate trial types. This suggests that the responses on trial types were stable over time. This might reflect stable attentional tendencies within participants (responding equally fast/slow to trials of the same trial type), it might however also reflect stable differences in overall reaction time across participants (i.e., participants who tend to react faster at baseline also have reacted faster at post-test). Whereas weak test-retest reliability of the indices might indicate that the OOOT was not sensitive to consistently capture AB, it might also be that these results are related to the fact that this study tested a non-clinical sample. That is, AB might have been unstable within participants, perhaps because AB to alcohol was not a distinct characteristic of the current student sample (see for example [37]). The test-retest reliability might also be affected by practice effects; indeed, participants became generally faster from baseline to post-test. However, there was no evidence that the strength of the practice effects varied across trial types, thus there was no straightforward evidence suggesting that differential practice effects might have contributed to differences in the relative strength of the bias measures between baseline and post-test. Future research should investigate the test-retest reliability of the current tasks in a sample that is expected to show a more stable and systematically occurring AB for alcohol (e.g., a clinical sample).

Research in several psychopathologies indicated the importance of using a task that is able to differentiate between attentional engagement and disengagement, as different disorders might be characterised by either or both of these attentional processes, which in turn might result in different indications for treatment [20,24]. Previous research on AB for alcohol have shown inconsistent results. One important reason for these inconsistencies might be the use of tasks that are unable to properly distinguish between these attentional processes [25,38]. The findings of the current study provide tentative support for the usefulness of distinguishing between engagement and disengagement processes in alcohol AB. That is, the OOOT findings indicated that frequency and quantity of alcohol consumption were related with difficulty to disengage attention but not attentional engagement. Further, the disengagement index continued to be related with the alcohol quantity measure when controlling for both the engagement index and the VST. AB for alcohol might therefore (mainly) be characterised by more difficulty to disengage attention from alcohol cues rather than increased engagement with these cues. Although the disengagement index appears to be more strongly related to alcohol use in the overall sample, post-hoc moderation analyses suggested that attentional engagement is related to frequency of consumed alcohol in males. Future studies should therefore further investigate the role of engagement and disengagement processes of attention in relation to alcohol consumption in a clinical sample, possibly separately looking at male and female participants. Knowing the attentional pattern in clinical samples can help improve current investigations testing the effectiveness of AB modification interventions. That is, AB modification interventions could then be especially designed to target the relevant processes.

Based on previous studies, one would expect AB indices to be related to craving [3,4], as craving has been described to have a circular relationship with AB for alcohol (i.e., bias-craving-bias cycle). Measures of craving were both unrelated to the VST and the OOOT. A possible explanation for these null findings is that the current sample consisted of non-clinical participants. The current sample of students showed generally low levels of alcohol craving, as well as generally low levels of alcohol use problems. A recent study showed that alcohol consumption in students is mainly associated with activities of students’ life [37]. Thus, drinking alcohol might predominantly come as a by-product of these activities rather than from the urge to drink. Therefore, AB might play a less pronounced role in the student population. In a clinical sample however, craving is expected to be more prominent. The association between AB and craving might therefore be a specific characteristic of problematic alcohol use, and less related to social drinking. However, given that recent studies in clinical samples also failed to find an association between AB and craving [12,39], it might also be that insufficient internal consistency and test-retest reliability of AB tasks limited their capacity to appropriately capture AB, making it difficult to find the proposed relation with craving.

Although the study has several strengths, including a large sample size, the inclusion of two assessment tasks that have not been used previously to assess AB for alcohol, and the repeated assessment allowing to examine stability of the AB measures, there are also several limitations that bear on the interpretation of the study results. First, in the current study we used a convenience sample of students to test the VST and the OOOT in the context of alcohol. There are indications that students’ motivation to drink alcohol might differ from adults. That is, alcohol consumption in students has been found to be associated with specific activities [37], and drinking alcohol might therefore be less related with AB. Future research should examine the validity (and reliability) of the current tasks in a clinical sample. Second, and in line with the previous limitation, the ratio of male and female participants was 1:4. Although male and female participants did not differ in the quantity and frequency of alcohol consumption, or the degree of reported craving or alcohol problems, the uneven distribution in gender should be kept in mind when interpreting the results. Especially because first investigations have shown that cognitive biases might only be positively related with alcohol consumption in male participants [33,34]. However, future well-powered studies are necessary to further disentangle the role of gender in AB. Third, the wording used in the MATE-Q is slightly different when asking about the quantity compared to the frequency of alcohol use. Whereas the quantity of alcohol use is asked to be reported on a ‘typical’ drinking day, the frequency of alcohol consumption is given for the number of days in the past 30 days without referring to ‘typical’ drinking days. We do not expect that this discrepancy in wording has had a substantial influence on the findings, however, some participants might slightly adopt their answers due to the addition of the word ‘typical’ (e.g., imagine a student who normally drinks two standard units of alcohol during one occasion, might not report the single day throughout the past 30 days in which she drank ten units during the marriage of her sister). Fourth, for the calculation of AB indices of the OOOT the two types of contrast categories (i.e., non-alcoholic drinks and flowerpots) were used as ‘neutral’ (i.e., non-alcohol) comparison categories that contrast with the category of interest (i.e., alcohol). However, it cannot be ruled out that the sensitivity of the task as an index of AB might differ as a function of the contrast category. It would be interesting for future research to test if the reliability and criterion validity of the AB measure might profit from using more easily identifiable contrast stimuli. To further investigate the specific effects of contrast categories when assessing AB, future studies might add a third contrast category to disentangle whether AB towards alcohol can be reliably measured when contrasted against content-related stimuli (i.e., non-alcoholic drinks) or non-content-related stimuli (e.g., flowerpots), and whether AB is specifically related to alcoholic drinks or drinks in general (by separately comparing alcohol stimuli and non-alcoholic drinks stimuli with the two other contrast categories; see for example [40]). Last, as the error rate of the relevant trials of the OOOT was found to be high, we had to exclude a substantial number of incorrect trials from the analyses. This might have reduced its reliability as a measure of individual differences. In comparison with the VST, the OOOT is more complex and difficult given that participants do not know whether or not an odd-one-out is present. In the future, the OOOT might therefore be improved by adding practice trials, and by providing participants with feedback (e.g., right or wrong) about their performance. Participants might then become more aware of how to differentiate between the stimuli, especially between the images of alcoholic versus non-alcoholic drinks.

## Conclusion

The current study revealed that the internal consistency and test-retest reliability of the AB indices of the VST and the OOOT are not optimal, but the findings are in line with previous results of the reliability of other reaction time tasks indexing AB, such as the visual probe task. Given the more adequate reflection of the complexity of real-life substance use relevant situations, the tasks might be preferred over the visual probe task that has been the task of choice until today. Further, the current study shows that it seems relevant to distinguish between engagement and disengagement processes of attention, as only the disengagement index was associated with both quantity and frequency of alcohol use. Therefore, it seems advisable to generally include a third (neutral) stimulus category within visual search tasks including the VST, because this allows to separately compute engagement and disengagement indices of AB. To further investigate the role of engagement and disengagement processes of attention, the addition of a more direct measure of AB might also be relevant, for example based on eye-tracking procedures. In sum, both visual search tasks showed some merit as a measure of AB for alcohol. However, before improved versions of these tasks can be recommended as measures of AB within the realm of substance use, it would be important to first verify the psychometric properties of these tasks in a clinical population.

## Supporting information

S1 File Relation between attentional bias measures and outcome measures when including non-users to the analyses. (DOCX)

S1 Table Correlations between attentional bias indices as measured with the VST and the OOOT. VST AB = index of attentional bias measured with the Visual Search Task; OOOT E = index of engagement as measured with the Odd-One-Out Task; OOOT D = index of disengagement as measured with the Odd-One-Out Task; *p< .05.
(DOCX)
