# Supplementary material for: Attentional bias for alcohol cues in visual search—Increased engagement, difficulty to disengage or both?
Source: PLoS One. 2020 Jan 27;15(1):e0228272. doi: 10.1371/journal.pone.0228272 (PMC6984682; doi:10.1371/journal.pone.0228272)
Supplement: S1 File — (DOCX) [file pone.0228272.s001.docx]

When including the non-drinkers to this analysis, the pattern remained the same. The VST continued to show a weak but significant relationship with frequency of alcohol use (*r* = .18, *p* = .025). For the OOOT, the disengagement index continued to show a weak association with the quantity (*r* = .19, *p* = .012) and frequency of alcohol use (*r* = .20, *p* = .012).
