# Supplementary material for: Attentional bias for alcohol cues in visual search—Increased engagement, difficulty to disengage or both?
Source: PLoS One. 2020 Jan 27;15(1):e0228272. doi: 10.1371/journal.pone.0228272 (PMC6984682; doi:10.1371/journal.pone.0228272)
Supplement: S1 Table — VST AB = index of attentional bias measured with the Visual Search Task; OOOT E = index of engagement as measured with the Odd-One-Out Task; OOOT D = index of disengagement as measured with the Odd-One-Out Task; *p< .05. (DOCX) [file pone.0228272.s002.docx]

|  | **VST AB** | **OOOT E** | **OOOT D** |
| --- | --- | --- | --- |
| VST AB | - | .08 | .02 |
| OOOT E | .08 | - | -.47* |
| OOOT D | .02 | -.47* | - |
